# Synthesis, characterization, and antifungal activity of chitosan–copper nanocomposites against crop pathogens

**DOI:** 10.3389/ffunb.2026.1764049

**Published:** 2026-03-17

**Authors:** A. D. Savalkar, P. R. Shingote, D. L. Wasule, A. M. Gaharwal, D. R. Rathod, S. S. Nichal, J. R. Katore, M. P. Moharil

**Affiliations:** 1 Vasantrao Naik Government College of Agricultural Biotechnology, Yavatmal, Dr. Panjabrao Deshmukh Krishi Vidyapeeth (PDKV), Akola, Maharashtra, India; 2 Biotechnology Centre, Department of Agriculture Botany, Dr. Panajbrao Deshmukh Krishi Vidyapeeth (PDKV), Akola, Maharashtra, India; 3 Regional Research Centre, Amravati, Dr. Panjabrao Deshmukh Krishi Vidyapeeth (PDKV), Akola, Maharashtra, India; 4 Krishi Vigyan Kendra, Selsura, Dr. Panjabrao Deshmukh Krishi Vidyapeeth (PDKV), Akola, Maharashtra, India

**Keywords:** antifungal activity, chickpea diseases, chitosan–copper nanoparticles, citrus diseases, nanobiopesticides, nanocomposite, plant pathogenic fungi, sustainable crop protection

## Abstract

Chitosan–copper nanoparticles (CHT–Cu NPs) were synthesized using an ionic gelation approach and evaluated for their physicochemical properties and antifungal activity against major fungal pathogens of chickpea and citrus. For instance, “In recent years, nanotechnology-based formulations have emerged as promising strategy for sustainable disease management”. Dynamic light scattering analysis revealed uniformly sized nanoparticles (~150 nm) with low polydispersity and a positive surface charge (+22.2 mV). Fourier transform infrared spectroscopy, X-ray diffraction, scanning and transmission electron microscopy, and energy-dispersive X-ray analysis confirmed effective copper coordination, amorphous nanocomposite formation, and stable incorporation of copper within the chitosan matrix. The antifungal efficacy of CHT–Cu NPs was assessed *in vitro* against *Colletotrichum ciceri*, *Fusarium ciceri*, *Rhizoctonia bataticola*, *Sclerotium rolfsii*, and *Colletotrichum gloeosporioides*. The nanocomposites exhibited strong, concentration-dependent inhibition of mycelial growth. *F. ciceri* was highly sensitive, showing complete inhibition at all tested concentrations (≥100 µg mL^−^¹). *S. rolfsii* and chickpea pathogens *C. ciceri* and *R. bataticola* were completely inhibited at concentrations 200 µg mL^−^¹, 300 µg mL^−^¹ and 300 µg mL^−^¹, respectively, whereas *C. gloeosporioides* was comparatively less sensitive and required higher concentrations (≥400 µg mL^−^¹) for complete suppression. In contrast, chitosan alone and copper sulfate showed only moderate antifungal activity. These findings demonstrate that CHT–Cu NPs possess broad-spectrum antifungal activity and superior efficacy compared to conventional fungicides, highlighting their potential as eco-compatible nanobiopesticides for sustainable management of fungal diseases in crop plants.

## Highlights

- Chitosan–copper nanoparticles were synthesized using a controlled ionic gelation method.
- Nanocomposites exhibited uniform nanoscale size (~150 nm), positive surface charge (+22.2 mV), and stable copper incorporation.
- CHT–Cu nanoparticles showed strong, concentration-dependent antifungal activity against major chickpea and citrus pathogens.
- Complete inhibition of *Fusarium ciceri* (100 µg mL^−^¹) and *Sclerotium rolfsii* (200 µg mL^−^¹) was achieved at relatively low concentrations.
- Chitosan–copper nanoparticles outperformed chitosan, copper sulfate, and a commercial fungicide *in vitro*.

## Introduction

Fungal pathogens are among the most destructive biotic stress factors affecting global agricultural productivity (Zhou et al., 2024; Wang et al., 2025). They cause a wide spectrum of diseases such as wilts, root rots, anthracnose, and collar rots, leading to substantial yield and quality losses in economically important crops (Strange and Scott, 2005; Dean et al., 2012; Williamson-Benavides and Dhingra, 2021). Chickpea (*Cicer arietinum* L.) and citrus (*Citrus* spp.) are major crops that play a vital role in food security and farm income, particularly in India; however, their production is frequently constrained by soil- and foliar-borne fungal diseases that are difficult to manage using conventional approaches (Pande et al., 2010; Timmer et al., 2000; Sankar et al., 2024; Chaudhary et al., 2025).

In chickpea, *Rhizoctonia bataticola*, *Colletotrichum capsici*, and *Fusarium ciceri* are major fungal pathogens responsible for dry root rot, anthracnose, and wilt, respectively (Mageshwaran et al., 2022). *F. ciceri* is particularly destructive, causing up to 80–100% yield loss under favorable conditions and persisting in soil through long-lived chlamydospores, which limits the effectiveness of crop rotation and chemical control (Jimenez-Diaz et al., 2015). Similarly, *R. bataticola* has become increasingly problematic under conditions of high temperature and moisture stress (Sharma and Pande, 2013), while *C. capsici* severely affects aerial plant parts, reducing seed quality and yield (Rani et al., 2020). In citrus, *Colletotrichum gloeosporioides* and *Sclerotium rolfsii* cause anthracnose and collar rot, respectively, and their survival in soil and plant debris further complicates disease management (Timmer et al., 2000; De Oliveira Costa et al., 2019).

Chemical fungicides, including copper- and azole-based formulations, remain the primary tools for managing these diseases. However, prolonged and indiscriminate use has contributed to the emergence of resistant fungal populations (Hahn, 2014), contamination of soil and water resources, adverse effects on non-target microflora, and concerns related to human health (Carvalho, 2017). In particular, repeated application of copper-based fungicides can result in copper accumulation in agricultural soils, raising concerns regarding phytotoxicity and ecological imbalance (Komarek et al., 2010; Kakutey et al., 2023; Mallano et al., 2023; Kos et al., 2026). These limitations have stimulated interest in alternative strategies that improve antifungal efficacy while reducing excessive chemical inputs.

Nanotechnology offers new opportunities for crop protection through the development of nanomaterials with improved stability, controlled delivery, and enhanced antimicrobial performance (Rai and Ingle, 2012; Chen and Yada, 2011; Jali et al., 2024; Vishnu et al., 2024). Among these, chitosan-based nanocomposites have received considerable attention. Chitosan is a biodegradable polysaccharide derived from chitin that exhibits intrinsic antimicrobial activity against a broad range of plant pathogens and has been widely explored as a carrier matrix for metal-based agents (Badawy and Rabea, 2011; El Hadrami et al., 2010). Its amino and hydroxyl functional groups facilitate strong interactions with metal ions, enabling stabilization and dispersion of metal nanoparticles within a polymeric matrix (Negm et al., 2020; Torkaman et al., 2021; Bian et al., 2022; Ul-Islam et al., 2024).

Copper is a well-established broad-spectrum antimicrobial agent; however, its conventional bulk or ionic forms present limitations related to persistence and non-target effects (Fagnano et al., 2020; Burandt et al., 2024; Su et al., 2024). Incorporation of copper into chitosan matrices at the nanoscale has been reported to enhance dispersion, improve stability, and enable controlled copper availability (Jo et al., 2009; Dutta et al., 2009). Several studies have demonstrated antifungal activity of chitosan–copper and related chitosan–metal nanocomposites against phytopathogenic fungi (Usman et al., 2020; Niu et al., 2021; Vanti et al., 2020; Saharan et al., 2015; Mosa and El-Abeid, 2023; Fatima et al., 2024; Munir et al., 2024). However, most reports remain restricted to single or model pathogen systems, rely largely on agar-based inhibition assays, or lack comprehensive integration of physicochemical characterization, copper release behavior, and quantitative fungicidal benchmarks such as minimum inhibitory and fungicidal concentrations (MIC/MFC). Direct comparison with chitosan alone, copper salts, or commercial fungicides under identical experimental conditions is also frequently lacking.

Consequently, systematic evaluations of chitosan–copper nanocomposites against multiple field-relevant pathogens of economically important crops such as chickpea and citrus remain scarce. Moreover, copper release behavior from chitosan–copper nanocomposites is rarely quantified, despite its central role in antifungal performance. The absence of integrated assessment linking synthesis strategy, physicochemical properties, copper release characteristics, and quantitative antifungal benchmarks limits clear interpretation of the added value of chitosan–copper nanocomposite formulations over conventional copper-based treatments.

In this context, the present study synthesizes chitosan–copper nanocomposites using a controlled ionic gelation approach and integrates physicochemical characterization with *in vitro* copper release profiling and quantitative antifungal evaluation against major fungal pathogens of chickpea and citrus. Comparative assessment with chitosan alone, copper sulfate, and a commercial fungicide under identical conditions enables a realistic evaluation of antifungal efficacy and highlights the application-level relevance of the nanocomposite system.

## Materials

Different chitosan specifications were initially evaluated, and the optimum type was selected to ensure consistent and reproducible nanoparticle synthesis. Shrimp-derived chitosan (<150 kDa) was used based on its solubility and suitability for ionic crosslinking. The chitosan exhibited 7.4% moisture content, 0.4% ash, a pH of 7.37 (in 1% acetic acid), viscosity of 160 cP (1% solution; Brookfield viscometer), and a degree of deacetylation of 89.64%. Sodium tripolyphosphate (TPP) and copper sulfate pentahydrate (CuSO_4_·5H_2_O) were procured from HiMedia Laboratories Pvt. Ltd. (Mumbai, India). All reagents were of analytical grade, and solutions were prepared using double-distilled water.

## Methods

Pathogenic isolates of *Rhizoctonia bataticola*, *Colletotrichum capsici*, and *Fusarium ciceri* (infecting chickpea), and *Colletotrichum gloeosporioides* and *Sclerotium rolfsii* (infecting citrus) were obtained from the culture collection of the Department of Plant Pathology, College of Agriculture, Nagpur. Cultures were maintained on Potato Dextrose Agar (PDA) slants at 4 °C and sub cultured at monthly intervals (Pande et al., 2010; Sharma et al., 2021).

### Preparation of chitosan solution

A 0.2% (w/v) chitosan solution was prepared by dissolving chitosan powder in 1% (v/v) glacial acetic acid under continuous stirring overnight at room temperature. The pH was adjusted to 5.5 using 1 M NaOH to optimize amino group protonation (Kashyap et al., 2015; Badawy and Rabea, 2011). The solution was filtered through Whatman No. 1 filter paper to remove undissolved residues.

### Synthesis of chitosan–Cu nanocomposite

Chitosan–copper nanocomposites were synthesized by the ionic gelation method (Calvo et al., 1997; El-Wakil et al., 2015). The pre-prepared chitosan solution was stirred magnetically, and a 0.01 M copper sulfate solution was added dropwise to facilitate electrostatic interaction between Cu²^+^ ions and protonated chitosan. Subsequently, a 0.1% (w/v) TPP solution was added dropwise as a crosslinking agent. The appearance of turbidity indicated nanoparticle formation. The suspension was centrifuged at 12,000 rpm for 30 min, washed twice with distilled water to remove unbound ions, and freeze-dried to obtain the nanocomposite powder.

### Physicochemical characterization

#### Particle size and Zeta potential

The average particle size, polydispersity index (PDI), and surface charge (zeta potential) were determined using Dynamic Light Scattering (DLS) (Anton Paar India Pvt. Ltd. Litesizer 500) following the protocol of Kashyap (2017) and Maluin and Hussein (2020).

### Fourier transform infrared spectroscopy

FTIR spectra of chitosan, copper sulfate, and CHT–Cu nanocomposites were recorded in the range of 4000–400 cm^−^¹ (Bruker, Germany) to identify characteristic functional groups and binding interactions (El Hadrami et al., 2010; Badawy and Rabea, 2011).

### X-ray diffraction

XRD analysis was performed using an X-ray diffractometer (Rigaku, Japan) with Cu Kα radiation (λ = 1.5406 Å) at a scanning speed of 2°/min in the 2θ range of 10°–80° to confirm crystallinity and phase composition (Ali et al., 2021; Huang et al., 2011).

### Transmission electron microscopy

The morphology and particle size distribution of the nanocomposites were observed using TEM (JEOL JEM-2100). One drop of diluted suspension was placed on a carbon-coated copper grid and dried at room temperature before imaging (Rai and Ingle, 2012; Kashyap et al., 2015).

### *In-vitro* release of Cu

The *in-vitro* experiment was conducted to evaluate the amount of Cu has been released from CHT–Cu NPs by following the methodology given by Choudhary et al., 2017 with some modifications. Dried nanoparticles were dispersed in distilled water and incubated at room temperature. At 48, 96, 144, and 192 h, samples were centrifuged at 10,000 rpm for 10 min, and the supernatant was collected. The released copper content was quantified using a UV–Visible spectrophotometer. The experiment was conducted in three biological replications.

### Evaluation of antifungal activity

Antifungal activity was assessed using the poisoned food technique (Grover and Moore, 1962; Ali et al., 2021). The concentration range (100–500 µg mL^−^¹) was selected based on earlier reports on chitosan-based and chitosan–copper nanomaterials (Vanti et al., 2020; Saharan et al., 2015). Nanoparticles were incorporated into molten PDA before solidification. A 5 mm mycelial disc from actively growing cultures was placed at the center of each plate, and plates were incubated at 28 ± 2 °C for 5–7 days. Copper sulfate (280 µg mL^−^¹) served as a positive control, and PDA without treatment served as a negative control.

### Measurement of mycelial growth inhibition

Colony diameter was measured, and percent inhibition of mycelial growth was calculated using the formula: Inhibition (%) = ((*C*−*T*)/*C*) ×100

where C = colony diameter in control and T = colony diameter in treatment (Vincent, 1947).

### Determination of minimum inhibitory concentration and minimum fungicidal concentration

The minimum inhibitory concentration (MIC) of chitosan–copper nanoparticles (CHT–Cu NPs) against major fungal pathogens was determined using a broth-based assay (Wayne, 2008). Fungal inocula were prepared from actively growing cultures and introduced into suitable broth medium containing different concentrations of CHT–Cu NPs (100–500 µg mL^−^¹). The cultures were incubated at 28 ± 2 °C for 72 h. Fungal growth was assessed visually and by measuring turbidity (OD_600_). The MIC was defined as the lowest concentration of CHT–Cu NPs showing no visible fungal growth in the broth medium.

The minimum fungicidal concentration (MFC) was determined by sub culturing 10 µL of the aliquots from MIC assay tubes showing no visible growth onto PDA plates. The plates were incubated at 28 ± 2 °C for seven days and examined for colony formation. The plates were incubated for 7 days and MFCs were determined as the lowest CHT–Cu NPs concentrations which showed the maximum (100%) inhibition of the fungi. The *in-vitro* experiment was carried out with each treatment was replicated three times.

### Biotoxic impact of CHT–Cu NPs on chickpea plants under *in-vitro* conditions

Healthy and uniform seeds developed at Dr. Panjabrao Deshmukh Krishi Vidyapeeth, Akola Maharashtra India were surface sterilized using 0.1% mercuric chloride solution for 2 min followed by 4–5 rinses with sterile distilled water. Sterilized seeds were aseptically inoculated into plant tissue culture bottles containing semi-solid half-strength Murashige and Skoog medium (½ MS) medium. The medium was supplemented with CHT–Cu NPs suspensions at concentrations of 100 µg mL^−^¹ and 800 µg mL^−^¹ before solidification. Bottles without nanoparticles served as the control. Each treatment consisted of 5 seeds and was maintained in triplicate. Cultures were incubated at 25 ± 2 °C under a 16 h light/8 h dark photoperiod for 7 days. Observations on seed germination were recorded daily. root length (cm), shoot length (cm), visual phytotoxic symptoms (chlorosis, necrosis, growth inhibition) and Seedling Vigor Index (SVI) =Germination (%) × (Root length+Shoot length).

Following the *in vitro* germination assay, uniformly germinated seedlings from each treatment were carefully transferred to earthen pots (25 × 22 cm) containing approximately 3 kg of sterilized sandy clay loam soil mixed with farmyard manure and sand in a 2:1:1 ratio. The seedlings corresponded to treatments previously exposed to CHT–Cu nanoparticle suspensions (100–800 µg mL^−^¹), while untreated seedlings served as the control. Each treatment was maintained in triplicate under greenhouse conditions (25 ± 3 °C) in a Completely Randomized Design. Pots were irrigated regularly with distilled water, and no fertilizers or chemicals were applied during the experimental period. Observations on plant growth were recorded at 20 days after transfer (DAT), including survival percentage, root length, shoot length, visual phytotoxic symptoms (chlorosis, necrosis, growth inhibition etc). Data were expressed as mean ± standard deviation.

### Statistical analysis

Experiments were conducted using a completely randomized design (CRD). Percentage data were subjected to arcsine (angular) transformation prior to analysis. Transformed data were analyzed using analysis of variance (ANOVA) in SPSS software, and treatment means were compared using Duncan’s Multiple Range Test (DMRT) at *p* ≤ 0.05. Results are presented as mean ± standard error (SE) (Gomez and Gomez, 1984).

## Results

### Synthesis of chitosan–copper nanoparticles

Chitosan–copper nanoparticles (CHT–Cu NPs) were successfully synthesized using the ionic gelation method with sodium tripolyphosphate (TPP) as a crosslinking agent. Nanoparticle formation was visually indicated by a distinct color change of the reaction mixture from yellow to sky blue, consistent with previous reports on chitosan–copper nanoparticle synthesis via ionic gelation (Saharan et al., 2015; Gomes et al., 2021).

### Physicochemical characterization of CHT–Cu NPs

#### Particle size and zeta potential

**Figure 1:**
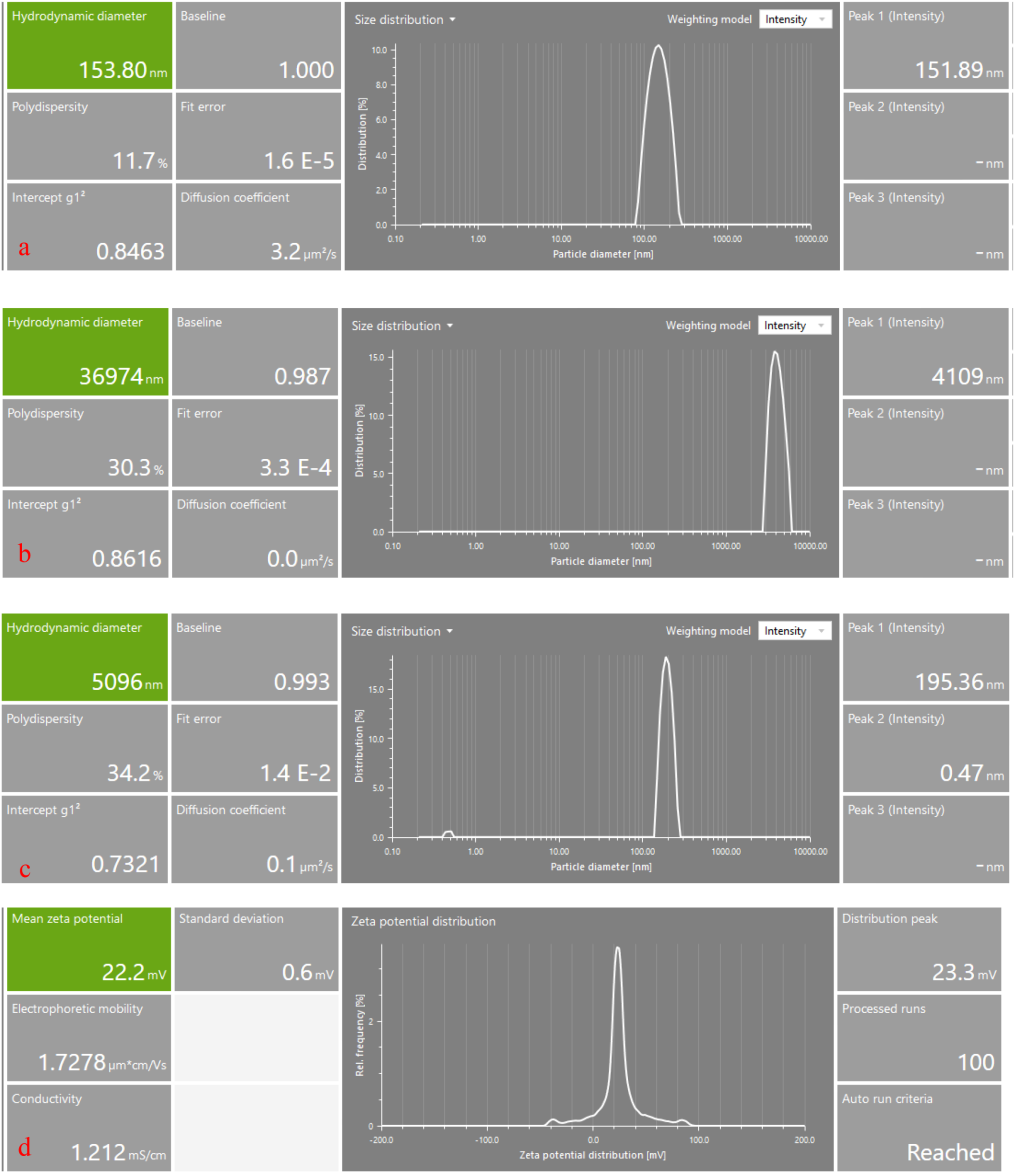
Dynamic light scattering (DLS) analysis of particle size distribution and zeta potential of chitosan–copper nanoparticles (CHT–Cu NPs). **(a)** Hydrodynamic diameter and size distribution of CHT–Cu NPs, **(b)** size distribution of native chitosan, **(c)** size distribution of CuSO_4_·5H_2_O, and **(d)** zeta potential distribution of CHT–Cu NPs. The nanocomposites showed a uniform nanoscale size (~150 nm) with low polydispersity and a positive surface charge, indicating good colloidal stability.

Dynamic light scattering (DLS) analysis revealed marked differences in hydrodynamic size distribution among native chitosan, CuSO_4_·5H_2_O, and synthesized CHT–Cu nanoparticles (**Figure 1**). The CHT–Cu NPs exhibited a mean hydrodynamic diameter of 153.8 nm with a dominant intensity peak at 151.89 nm (**Figure 1a**) and a low polydispersity index (PDI = 0.117), indicating a narrow and uniform size distribution.

In contrast, native chitosan showed a highly heterogeneous size distribution with a large hydrodynamic diameter (36,974 nm), reflecting polymer aggregation in aqueous solution (**Figure 1b**). CuSO_4_·5H_2_O exhibited a broad size distribution (5,096 nm) with a high PDI, indicating poor dispersion (**Figure 1c**).

Zeta potential analysis showed that CHT–Cu NPs carried a positive surface charge of +22.2 mV, with a major peak at +23.3 mV (**Figure 1d**). Electrophoretic mobility (1.7278 µm·cm/V·s) and conductivity (1.212 mS/cm) values further characterized the colloidal properties of the nanocomposites.

### FTIR spectroscopic analysis of CHT–Cu nanoparticles

**Figure 2:**
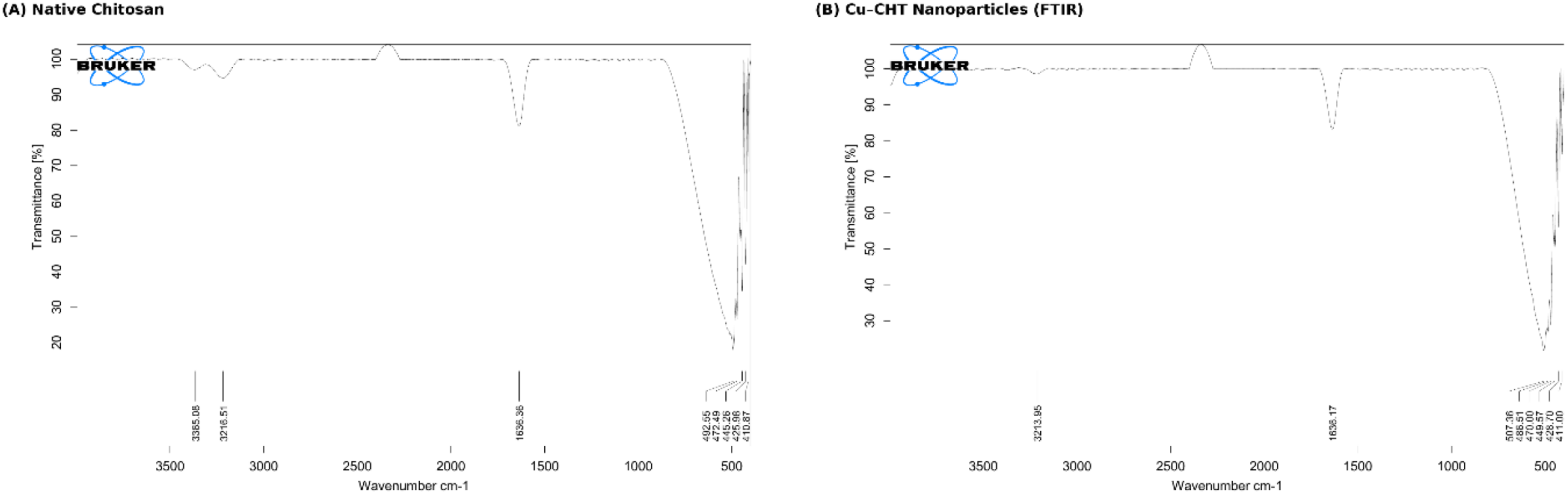
FTIR spectra of **(A)** native chitosan and **(B)** chitosan–copper nanoparticles (CHT–Cu NPs) showing band shifts after copper incorporation, confirming coordination of Cu²^+^ with chitosan functional groups and nanocomposite formation.

The FTIR spectra of pure chitosan and CHT–Cu nanoparticles are shown in **Figure 2**. Pure chitosan exhibited characteristic absorption bands, including a broad –OH/–NH stretching band at 3365.08 cm^−^¹, an N–H stretching band at 3216.51 cm^−^¹, and an amide I band at 1636.36 cm^−^¹, confirming its polysaccharide structure (**Figure 2a**).

In CHT–Cu nanoparticles, the –OH/–NH band shifted to 3213.95 cm^−^¹ with reduced intensity, while the amide I band showed a slight shift to 1636.17 cm^−^¹, indicating interaction between chitosan functional groups and Cu²^+^ ions. New absorption bands in the low-frequency region (507.36–411.00 cm^−^¹) corresponding to Cu–O and Cu–N stretching vibrations further confirmed chitosan–copper coordination (**Figure 2b**).

### X-ray diffraction analysis of CHT–Cu nanoparticles

**Figure 3:**
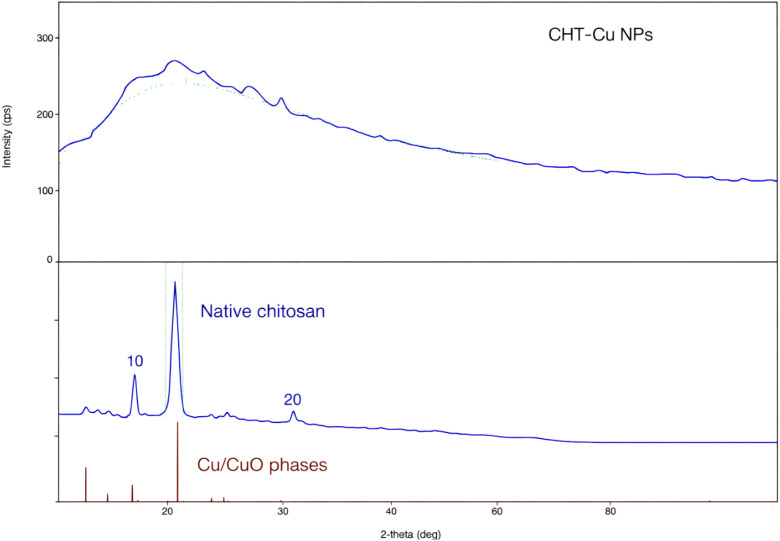
X-ray diffraction (XRD) pattern of chitosan–copper nanoparticles (CHT–Cu NPs) showing a predominantly amorphous structure with a broad diffraction halo, indicative of disrupted chitosan crystallinity and successful incorporation of copper within the polymeric matrix.

XRD patterns of native chitosan and CHT–Cu nanoparticles are presented in **Figure 3**. Native chitosan exhibited characteristic semi-crystalline peaks at approximately 2θ ≈ 10° and 20°. In contrast, CHT–Cu nanoparticles showed a broad diffraction halo in the range of 2θ ≈ 18–25°, with a marked reduction or disappearance of chitosan crystalline peaks, indicating a predominantly amorphous structure. No sharp peaks corresponding to crystalline copper or copper salt phases were detected.

### Morphological characterization of CHT–Cu nanoparticles

**Figure 4:**
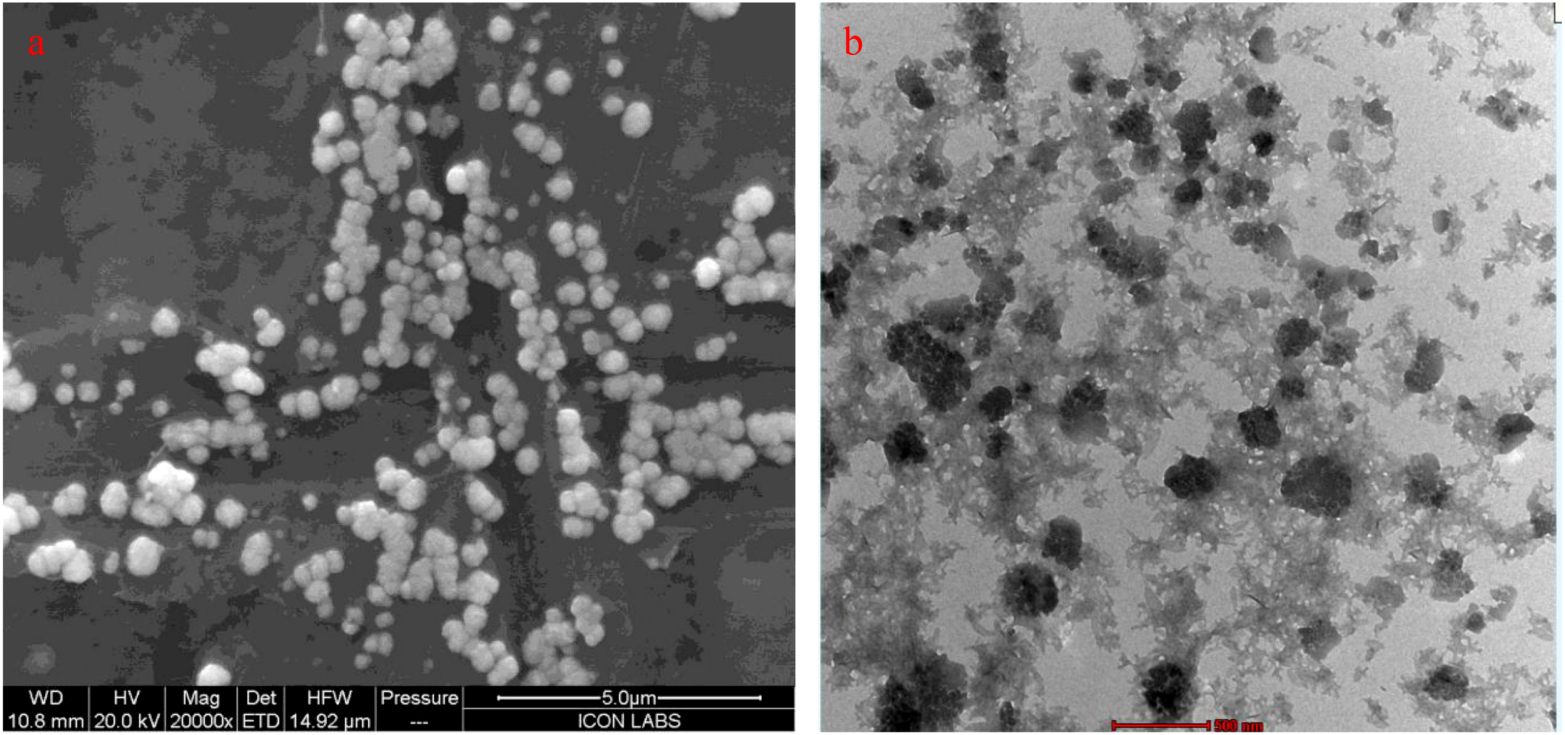
Morphological and elemental characterization of synthesized CHT–Cu nanoparticles. **(a)** FESEM micrograph showing spherical nanoparticles uniformly distributed on the chitosan matrix. **(b)** TEM image confirming nanosized CuO particles (250–320 nm) embedded in a porous chitosan–TPP network.

The morphology and structural features of the synthesized chitosan–copper nanoparticles (CHT–Cu NPs) were examined using scanning electron microscopy (SEM) and transmission electron microscopy (TEM) (**Figure 4**).

SEM micrographs (**Figure 4a**) revealed that the CHT–Cu NPs were predominantly spherical to near-spherical in shape with relatively uniform size distribution (200–650 nm). The nanoparticles appeared as discrete entities with limited agglomeration, indicating effective stabilization by the chitosan matrix. The surface morphology was smooth to slightly rough, characteristic of polymer-based nanocomposites formed through ionic gelation.

TEM analysis (**Figure 4b**) provided further confirmation of nanoparticle formation at the nanoscale. The nanoparticles appeared as well-dispersed, electron-dense cores embedded within a lighter polymeric matrix, confirming successful incorporation of copper within the chitosan framework. The particle size (250–320 nm) observed under TEM broadly corresponded with the hydrodynamic diameter obtained from DLS analysis, although individual particles appeared smaller due to dehydration and absence of solvation layers during TEM sample preparation.

### Elemental composition analysis by EDAX

Energy-dispersive X-ray analysis (EDAX) confirmed the elemental composition of the synthesized nanocomposites (**Table 1**). Copper was the dominant element, accounting for 75.43 wt% (29.48 at%), indicating high copper loading within the nanocomposite. Carbon (10.89 wt%), nitrogen (10.60 wt%), and oxygen (3.08 wt%) were also detected, corresponding to the chitosan polymer backbone.

**Table 1:** Elemental composition of chitosan–copper nanoparticles (CHT–Cu NPs) determined by Energy Dispersive X-ray Analysis (EDAX), showing dominant copper content along with carbon, nitrogen, and oxygen derived from the chitosan matrix.

Element	Wt%	At%
*Cu*	75.43	29.48
*C*	10.89	36.50
*N*	10.6	30.02
*O*	3.08	4.0
*Matrix*	Correction	ZAF

The coexistence of Cu with C, N, and O confirms the formation of a coordinated chitosan–copper nanocomposite rather than a simple physical mixture. The absence of extraneous elemental peaks indicates good purity of the synthesized material and effective removal of unbound components.

The collective physicochemical analyses confirmed the formation of stable, nanoscale chitosan–copper nanoparticles with uniform morphology and effective copper incorporation. Based on these validated structural properties, the antifungal activity of CHT–Cu NPs was subsequently evaluated.

### *In-vitro* release of Cu

The *in vitro* copper release profile of CHT–Cu nanoparticles is shown in **Supplementary Table 1**. Approximately 35% copper release was observed at 48 h, increasing to about 65% at 96 h. A gradual increase was observed thereafter, reaching approximately 68% and 70% at 144 h and 192 h, respectively (**Supplementary Table 1**). The release profile indicates an initial moderate release followed by sustained copper availability over time.

#### Effect on chickpea pathogens

**Figure 5:**
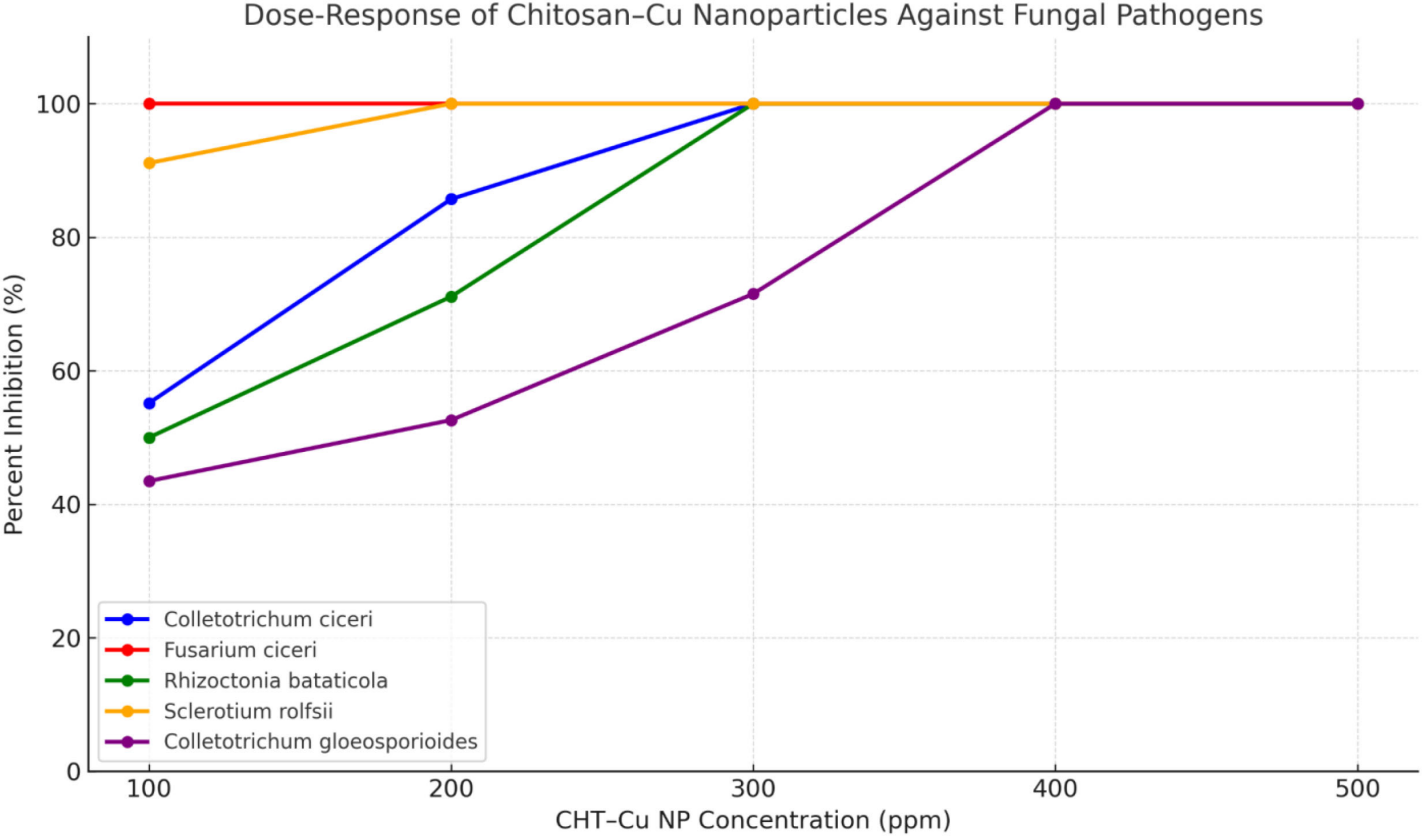
Percent inhibition of chickpea (*Colletotrichum ciceri*, *Fusarium ciceri*, *Rhizoctonia bataticola*) and citrus (*Sclerotium rolfsii*, *Colletotrichum gloeosporioides*) pathogens at 7 days after inoculation with chitosan–Cu nanoparticles (CHT–Cu NPs) at 100–500 µg mL^−^¹. Data represent mean ± SE of three replicates. Different letters indicate significant differences at *p* ≤ 0.05 (DMRT). Complete inhibition (100%) was observed at concentrations ≥200 µg mL^−^¹ for most pathogens except *Colletotricum gloeosporioides*.

Against *Colletotrichum ciceri*, CHT–Cu NPs caused 55.16% inhibition at 100 µg mL^−^¹, which increased significantly to 85.71% at 200 µg mL^−^¹. Complete inhibition (100%) was achieved at 300 µg mL^−^¹ and maintained at higher concentrations (**Tables 2**, **3**; **Figure 5**). These inhibition levels were significantly higher than those recorded for chitosan alone (0.4% 4000 µg mL^−^¹), CuSO_4_·5H_2_O (280 µg mL^−^¹), and Bavistin 500 µg mL^−^¹, which showed only limited to moderate suppression.

**Table 2:** Broth-based determination of minimum inhibitory concentration (MIC) of chitosan–copper nanoparticles (CHT–Cu NPs) against major fungal pathogens.

Pathogen	MIC (µg mL^−^¹)	Fungal growth at MIC	OD_600_/visual turbidity
*Colletotrichum ciceri*	300	−	No visible growth
*Fusarium ciceri*	100	−	No visible growth
*Rhizoctonia bataticola*	300	−	No visible growth
*Colletotrichum gloeosporioides*	400	−	No visible growth
*Sclerotium rolfsii*	200	−	No visible growth

MIC defined as the lowest concentration showing no visible fungal growth in broth medium.

**Table 3:** *In vitro* antifungal activity of chitosan–copper nanoparticles (CHT–Cu NPs) against major chickpea and citrus pathogens at seven days after inoculation.

Treatments	Percent Inhibition (%) at 7 days after inoculation				*Colletotrichum gloeosporioides*
	*Colletotrichum ciceri*	*Fusarium ciceri*	*Rhizoctonia bataticola*	*Sclerotium rolfsii*	
CHT Cu NPs @ 100 µg mL^−^¹	55.16 ± 0.19^b^	100	50 ± 0.14^b^	91.11 ± 0.42^a^	43.48 ± 0.04^c^
CHT Cu NPs @ 200 µg mL^−^¹	85.71 ± 0.06^a^	100	71.11 ± 0.31^a^	100	52.61 ± 0.2^b^
CHT Cu NPs @ 300 µg mL^−^¹	100	100	100	100	71.52 ± 0.13^a^
CHT Cu NPs @ 400 µg mL^−^¹	100	100	100	100	100
CHT Cu NPs @ 500 µg mL^−^¹	100	100	100	100	100
Chitosan @ 4000 µg mL^−^¹	26.11 ± 0.19^d^	28.15 ± 0.07^b^	26.11 ± 0.25^d^	11.11 ± 0.83^c^	22.17 ± 0.08^e^
Bavistin @ 500 µg mL^−^¹	46.63 ± 0.31^c^	50 ± 0.25^a^	38.52 ± 0.37^c^	45.37 ± 1.37^b^	38.04 ± 0.25^d^
CuSO_4_.5H_2_O @ 280 µg mL^−^¹	36.11 ± 0.19^d^	38.15 ± 0.07^b^	36.11 ± 0.25^d^	11.11 ± 0.83^c^	32.17 ± 0.08^e^
Control	–	–	–	–	–

All values are Mean ± SE.

*Fusarium ciceri* was the most sensitive chickpea pathogen tested, exhibiting complete inhibition (100%) at the lowest nanoparticle concentration of 100 µg mL^−^¹. This level of inhibition was sustained across all higher concentrations (200–500 µg mL^−^¹) and was statistically superior to the inhibition achieved by chitosan, copper sulfate, and Bavistin (**Table 3**; **Supplementary Figure 1b**).

For *Rhizoctonia bataticola*, moderate inhibition (50.00%) was observed at 100 µg mL^−^¹, which increased to 71.11% at 200 µg mL^−^¹. Complete inhibition (100%) was achieved at 300 µg mL^−^¹ and maintained at higher concentrations (**Table 3**; **Figure 5**). Lower nanoparticle concentrations (100–200 µg mL^−^¹) were also significantly more effective than the corresponding control treatments. Overall, chickpea pathogens differed in their sensitivity to CHT–Cu NPs, with *F. ciceri* showing the highest sensitivity, followed by *C. ciceri* and *R. bataticola*.

#### Effect on citrus pathogens

*Colletotrichum gloeosporioides* showed comparatively lower sensitivity at lower nanoparticle concentrations, with inhibition values of 43.48% and 52.61% at 100 and 200 µg mL^−^¹, respectively. Inhibition increased to 71.52% at 300 µg mL^−^¹, while complete suppression was achieved only at higher concentrations (400 and 500 µg mL^−^¹) (**Table 3**; **Figure 5**). At all concentrations, CHT–Cu NP treatments resulted in significantly higher inhibition than chitosan alone, CuSO_4_·5H_2_O, and Bavistin.

In contrast, *Sclerotium rolfsii* exhibited high sensitivity to the nanocomposite formulation. Inhibition reached 91.11% at 100 µg mL^−^¹, and complete inhibition (100%) was observed at 200 µg mL^−^¹ and maintained at higher concentrations (**Table 3**). These inhibition levels were significantly greater than those achieved with the control treatments. Among the citrus pathogens tested, *S. rolfsii* was more sensitive to CHT–Cu NPs than *C. gloeosporioides*.

### Determination of minimum inhibitory concentration and minimum fungicidal concentration

Broth-based MIC assays demonstrated strong antifungal activity of CHT–Cu nanoparticles against all tested fungal pathogens, with MIC values ranging from 100 to 400 µg mL^−^¹ (**Table 2**). *Fusarium ciceri* was the most sensitive pathogen, exhibiting complete growth inhibition at 100 µg mL^−^¹, whereas *Sclerotium rolfsii* showed an MIC of 200 µg mL^−^¹. Higher concentrations were required to inhibit *Colletotrichum ciceri* and *Rhizoctonia bataticola* (MIC = 300 µg mL^−^¹), while *Colletotrichum gloeosporioides* was comparatively less sensitive, with an MIC of 400 µg mL^−^¹.

MFC determination through sub culturing from MIC assays confirmed the fungicidal nature of CHT–Cu nanoparticles (**Table 4**). For all tested pathogens, MFC values were identical to the corresponding MIC values, as no fungal growth or colony formation was observed on PDA plates at these concentrations. In contrast, chitosan alone, and copper sulfate, failed to achieve complete inhibition or fungicidal effects at the tested concentrations.

**Table 4:** Minimum fungicidal concentration (MFC) of chitosan–copper nanoparticles (CHT–Cu NPs) determined by sub culturing from MIC assays onto PDA.

Pathogen	MFC (µg mL^−^¹)	Growth on PDA	Colony formation
*Colletotrichum ciceri*	300	No	Nil
*Fusarium ciceri*	100	No	Nil
*Rhizoctonia bataticola*	300	No	Nil
*Colletotrichum gloeosporioides*	400	No	Nil
*Sclerotium rolfsii*	200	No	Nil

MFC defined as the lowest concentration showing no fungal growth upon sub culturing.

### Biotoxic impact of CHT–Cu NPs on chickpea plants under *in-vitro* conditions

The *in vitro* phytotoxicity assessment revealed that chitosan–copper nanocomposites did not adversely affect chickpea seed germination or early seedling growth across most tested concentrations (**Supplementary Table 13**; **Supplementary Figure 2**). Germination percentage remained comparable to the control (93.3%) up to 700 µg mL^−^¹, with a slight enhancement observed at 400 µg mL^−^¹ (100% germination). Similarly, the Seedling Vigor Index (SVI) showed values equal to or higher than the control across concentrations up to 700 µg mL^−^¹. A marginal reduction in germination (86.7%) and SVI (690.00) was observed only at the highest concentration tested (800 µg mL^−^¹). These observations indicate that the nanocomposite is largely phytocompatible within the effective antifungal concentration range and does not impose significant toxicity on chickpea seedlings under *in vitro* conditions. Root and shoot growth recorded at 20 days after transfer (DAT) were similar to those of control plants, indicating normal seedling establishment. Visual observations also did not reveal any phytotoxic symptoms such as chlorosis, necrosis, stunted growth, or morphological abnormalities in these treatments (**Supplementary Figure 3**). These results confirm that the tested nanocomposite concentrations are phytocompatible for chickpea growth control condition.

## Discussion

### Physicochemical attributes governing antifungal performance

The physicochemical properties of the synthesized chitosan–copper nanoparticles (CHT–Cu NPs) provide a strong foundation for their observed antifungal activity. Dynamic light scattering analysis confirmed a uniform nanoscale size (~150 nm), placing the nanoparticles within the optimal range reported for effective antimicrobial applications. Previous studies have demonstrated that chitosan-based nanoparticles sized between 100 and 300 nm show improved interaction with fungal cell walls due to higher surface area and enhanced adhesion to hyphal surfaces, resulting in increased biological efficacy (Kashyap et al., 2015; Maluin and Hussein, 2020).

The physicochemical properties of the synthesized chitosan–copper nanoparticles (CHT–Cu NPs) provide an important basis for interpreting their antifungal performance. Dynamic light scattering analysis confirmed a uniform nanoscale size (~150 nm), which falls within the range commonly reported for chitosan-based nanomaterials exhibiting biological activity (Kashyap et al., 2015; Maluin and Hussein, 2020). The marked reduction in particle size compared with bulk chitosan reflects effective ionic gelation and coordination between protonated amino groups of chitosan and copper ions, as reported previously for chitosan–metal nanocomposites (Bhumkar and Pokharkar, 2006; Bamgbose et al., 2014). The low polydispersity index (PDI = 0.117) indicates a relatively homogeneous nanoparticle population, which is considered desirable for reproducible antifungal activity (Clarke, 2013; Danaei et al., 2018). The positive surface charge (+22.2 mV) of CHT–Cu NPs arises from protonated amino groups of chitosan and is comparable to values reported for antifungal chitosan-based nanocomposites (Badawy and Rabea, 2011; Rubina et al., 2017). Similar cationic surface characteristics have been associated with improved colloidal stability and enhanced interaction with fungal cell surfaces in previous studies, which may contribute to antifungal activity without implying a specific mode of action (Krumova et al., 2024).

### Molecular and structural evidence of nanocomposite formation

FTIR analysis provided clear molecular-level evidence for the successful coordination of copper within the chitosan matrix. Shifts and broadening of the combined –OH/–NH stretching band, along with subtle modifications in the amide I region, indicate strong interactions between Cu²^+^ ions and amino, amide, and hydroxyl functional groups of chitosan. These spectral features are consistent with earlier reports identifying –NH_2_ groups as primary chelation sites in chitosan–metal nanocomposites (Jo et al., 2009; Rubina et al., 2017). The appearance of distinct low-frequency Cu–O and Cu–N stretching bands further confirms copper–chitosan coordination through a self-assembly process (Guibal, 2004; Varma et al., 2004).

X-ray diffraction analysis revealed a predominantly amorphous structure of CHT–Cu nanoparticles, characterized by the disappearance of native chitosan crystalline peaks. Such amorphization is commonly reported for chitosan–metal nanocomposites synthesized via ionic gelation or coordination routes, where strong polymer–metal interactions disrupt regular chain packing (Ismail et al., 2023). Similar structural transitions have been associated with enhanced antimicrobial performance of chitosan-based nanomaterials against phytopathogenic fungi, including *Fusarium*, *Rhizoctonia*, and *Colletotrichum* species.

SEM and TEM observations further confirmed nanoscale particle formation with predominantly spherical morphology and limited aggregation (Atanda et al., 2025). Electron-dense regions observed in TEM images indicate effective incorporation of copper within the polymeric matrix. Minor aggregation is typical of biopolymer-based nanoparticles during sample preparation and does not contradict the uniform size distribution observed in solution-based measurements.

EDAX analysis demonstrated high copper content along with carbon, nitrogen, and oxygen derived from the chitosan backbone, confirming formation of a coordinated polymer–metal nanocomposite rather than a physical mixture. Similar elemental compositions have been reported for chitosan–copper nanocomposites synthesized using green or ionic gelation approaches (Mohamed et al., 2024). Higher copper loading within chitosan matrices has been associated with enhanced antifungal activity through regulated copper availability, while the polymeric framework moderates potential adverse effects associated with conventional copper formulations.

### Copper release behavior and its relevance to antifungal activity

The *in vitro* copper release study demonstrated a time-dependent and sustained release profile from CHT–Cu nanoparticles, characterized by an initial release phase followed by gradual stabilization over time. Comparable release patterns have been reported for chitosan–metal nanocomposites prepared via ionic gelation, where the polymeric matrix regulates metal ion diffusion into the surrounding medium (Bagchi et al., 2013; Choudhary et al., 2017).

This controlled release behavior provides experimental support for prolonged copper availability rather than rapid leaching. Such sustained release is particularly relevant for antifungal applications, as it may allow continuous exposure of fungal pathogens to bioavailable Cu²^+^ ions over extended periods. Importantly, the copper release data complement the antifungal bioassays and support interpretation of the observed concentration-dependent inhibition in both agar-based and broth-based assays.

### Antifungal efficacy and pathogen-specific responses

CHT–Cu nanoparticles exhibited strong, concentration-dependent antifungal activity against all tested chickpea and citrus pathogens, as consistently demonstrated by agar-based growth inhibition assays and broth-based MIC/MFC determinations. The close agreement between these independent assay systems strengthens the reliability of the observed antifungal effects and supports a fungicidal outcome.

Among chickpea pathogens, *Fusarium ciceri* was the most sensitive species, showing complete inhibition at the lowest tested concentration in both assay formats. Similar sensitivity of *Fusarium* species to chitosan-based and copper-containing nanomaterials has been reported previously, although effective concentrations vary depending on formulation and experimental conditions (Vanti et al., 2020; El-Naggar et al., 2024; Iqbal et al., 2024; Kumar et al., 2024). The low MIC and MFC values obtained in the present study indicate that inhibition was fungicidal rather than merely suppressive.

*Colletotrichum ciceri* and *Rhizoctonia bataticola* required moderately higher concentrations for complete inhibition, reflecting pathogen-specific variability. Partial inhibition at lower doses followed by complete suppression at higher concentrations was observed consistently across agar and broth assays. Comparable dose–response relationships have been reported for *Rhizoctonia* species treated with chitosan–metal nanocomposites (Rubina et al., 2017; Chen et al., 2024; Abd El-Wahab et al., 2025).

Among citrus pathogens, *Sclerotium rolfsii* exhibited high susceptibility, with low MIC and MFC values and complete inhibition at relatively low concentrations, whereas *Colletotrichum gloeosporioides* required higher nanoparticle concentrations for full suppression. Similar interspecific variability has been documented previously and is commonly attributed to inherent differences in fungal physiology and cell wall composition (Saharan et al., 2015).

Although the present study did not directly investigate cellular or molecular mechanisms of fungal inhibition, the observed antifungal activity of CHT–Cu nanoparticles can be interpreted in light of established literature and the experimental evidence generated here. Chitosan is known to interact electrostatically with negatively charged fungal cell wall and membrane components, potentially altering surface integrity and growth dynamics (El Hadrami et al., 2010; Badawy and Rabea, 2011). Incorporation of copper within the chitosan matrix provides a sustained source of bioavailable Cu²^+^ ions, as confirmed by the release profile obtained in this study.

Copper ions have been reported to interfere with essential fungal cellular processes, including enzyme activity, redox balance, and membrane-associated functions (Jo et al., 2009; Saharan et al., 2015). In addition, the nanoscale size and positive surface charge of CHT–Cu nanoparticles may facilitate close contact with fungal hyphae, enhancing localized copper availability at the cell surface. The close correspondence between MIC and MFC values across pathogens further supports a fungicidal effect.

While the present study demonstrates strong *in vitro* antifungal activity supported by physicochemical characterization, copper release profiling, and quantitative MIC/MFC evaluation, the performance of CHT–Cu nanoparticles under plant and soil conditions was not assessed. Evaluation of efficacy, phytotoxicity, and environmental behavior under greenhouse and field conditions will be essential to validate their practical applicability, and such studies are planned as part of future work.

The phytotoxicity assessment under *in vitro* conditions showed that CHT–Cu nanocomposites did not adversely affect chickpea seed germination or early seedling growth within the effective antifungal concentration range. These findings suggest that the tested nanocomposite concentrations are largely phytocompatible and suitable for further evaluation as plant protection inputs, subject to detailed field-level safety validation.

## Conclusion

This study reports the successful synthesis of chitosan–copper nanoparticles with uniform nanoscale size, positive surface charge, and effective copper incorporation. The nanocomposites exhibited strong, concentration-dependent *in vitro* antifungal activity against major chickpea and citrus pathogens, achieving complete inhibition of most fungi at relatively low concentrations, while *Colletotrichum gloeosporioides* required higher doses. Compared with chitosan alone, and copper sulfate, CHT–Cu nanoparticles consistently showed superior antifungal efficacy. Further Phytotoxicity assessments under both *in vitro* and pot conditions indicated that concentrations up to 700 µg mL^−^¹ did not adversely affect seed germination or early seedling growth of chickpea. Integration of physicochemical characterization, copper release profiling, and MIC/MFC determination underscores the potential of chitosan–copper nanocomposites as effective antifungal agents, warranting further evaluation under greenhouse and field conditions.

## Data Availability

The original contributions presented in the study are included in the article/**Supplementary Material**. Further inquiries can be directed to the corresponding author.
