# Supplementary material for: Synthesis, characterization, and antifungal activity of chitosan–copper nanocomposites against crop pathogens
Source: Front Fungal Biol. 2026 Mar 17;7:1764049. doi: 10.3389/ffunb.2026.1764049 (PMC13036234; doi:10.3389/ffunb.2026.1764049)
Supplement: Supplementary file 1 [file Supplementaryfile1.docx]

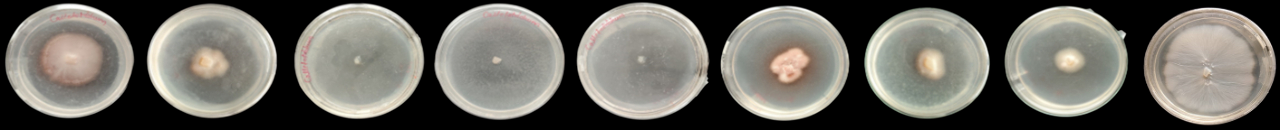


100 µg mL⁻¹

300 µg mL⁻¹

200 µg mL⁻¹

400 µg mL⁻¹

500 µg mL⁻¹

Chitosan

Bavistin

CuSO_4_.5H_2_O

Control

***Colletotrichum ciceri***


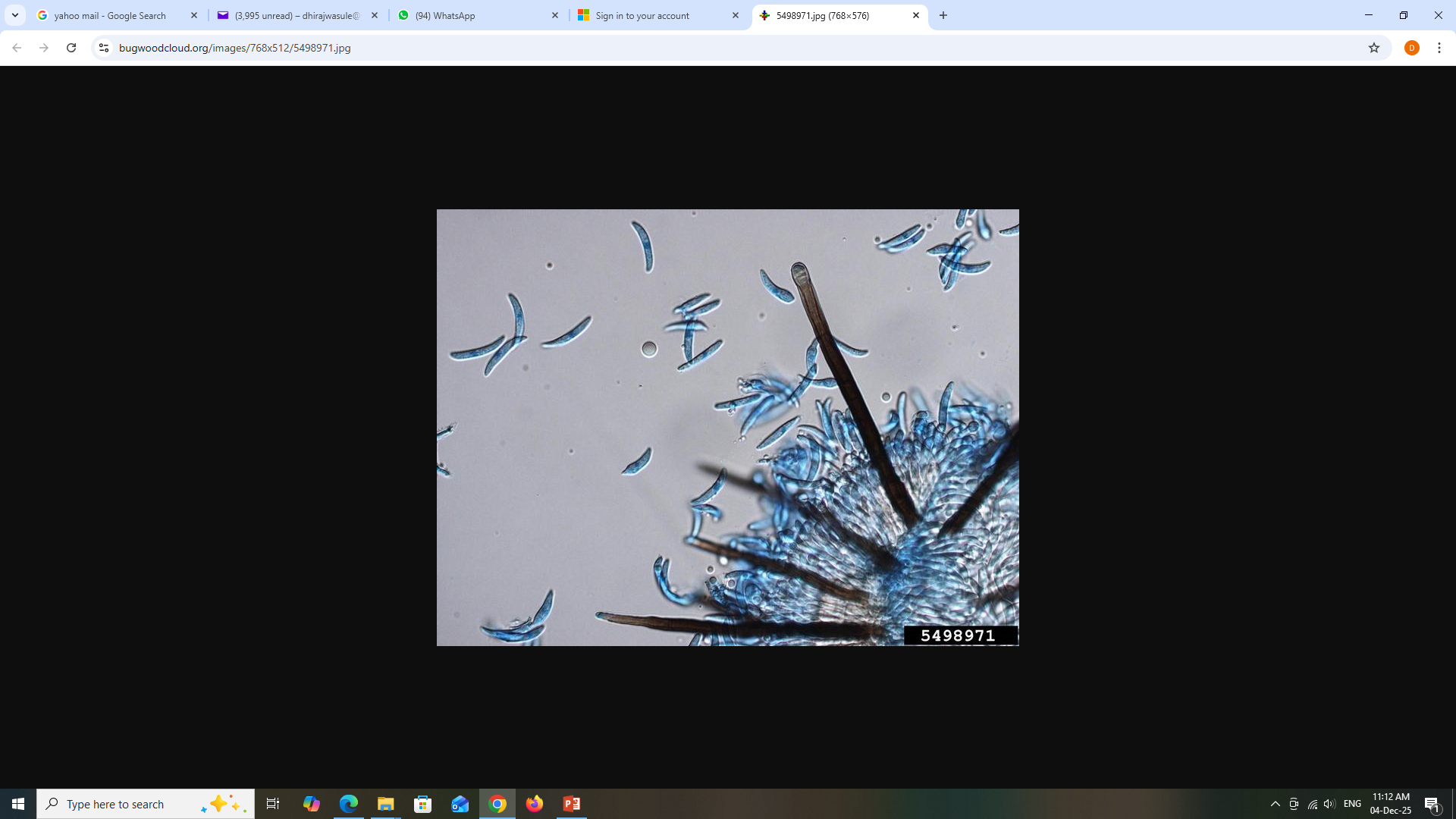


a


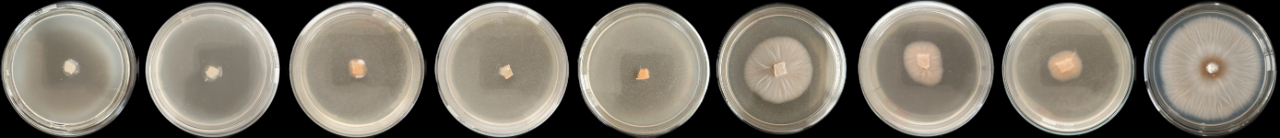


100 µg mL⁻¹

300 µg mL⁻¹

200 µg mL⁻¹

400 µg mL⁻¹

500 µg mL⁻¹

Chitosan

Bavistin

CuSO_4_.5H_2_O

Control

***Fusarium ciceri***


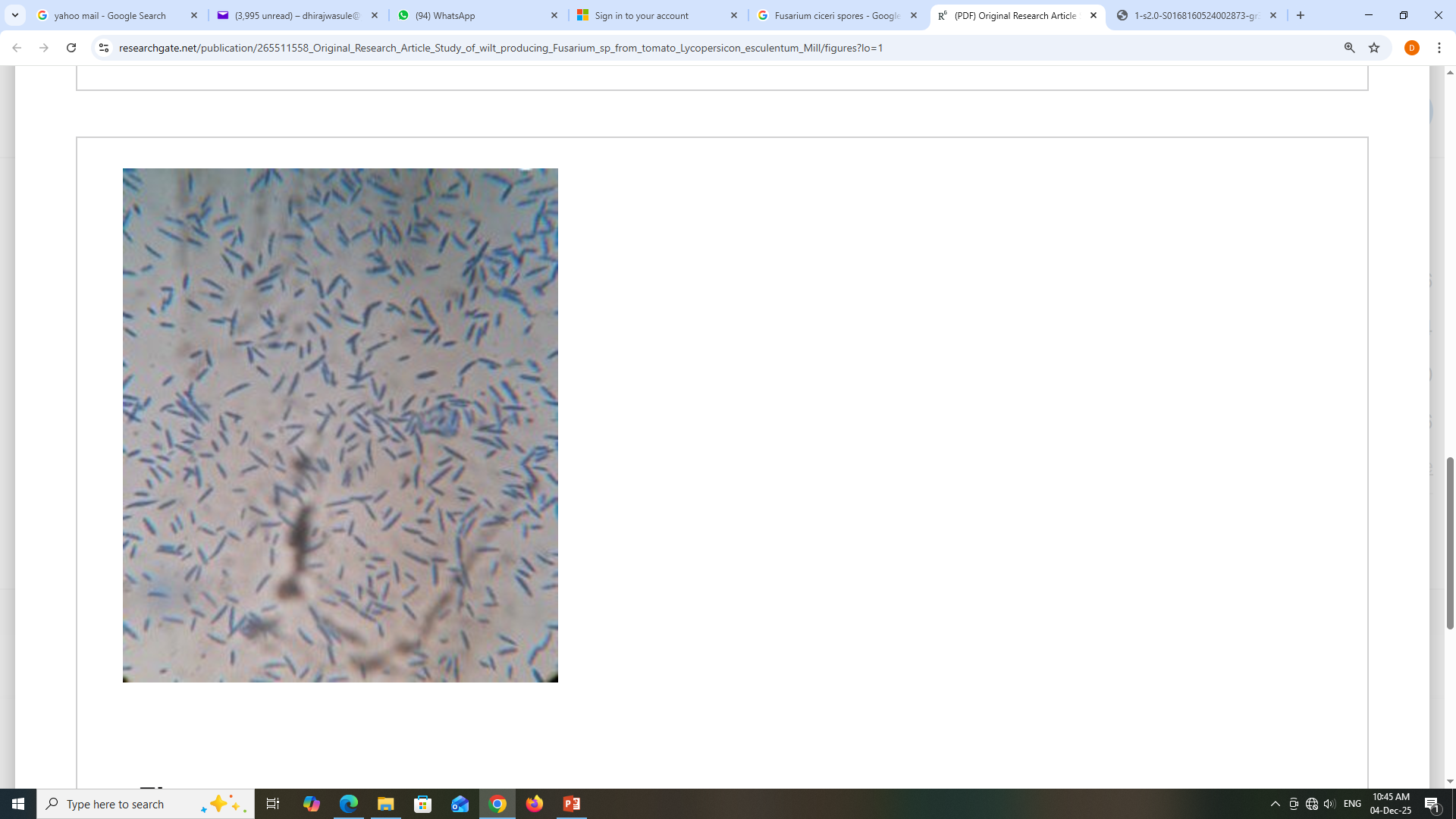


b


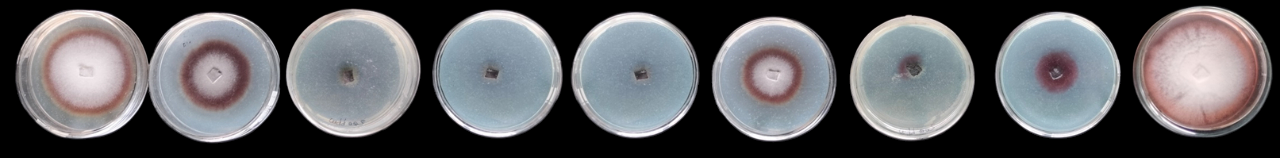


100 µg mL⁻¹

300 µg mL⁻¹

200 µg mL⁻¹

400 µg mL⁻¹

500 µg mL⁻¹

Chitosan

Bavistin

CuSO_4_.5H_2_O

Control

***Rhizoctonia bataticola***


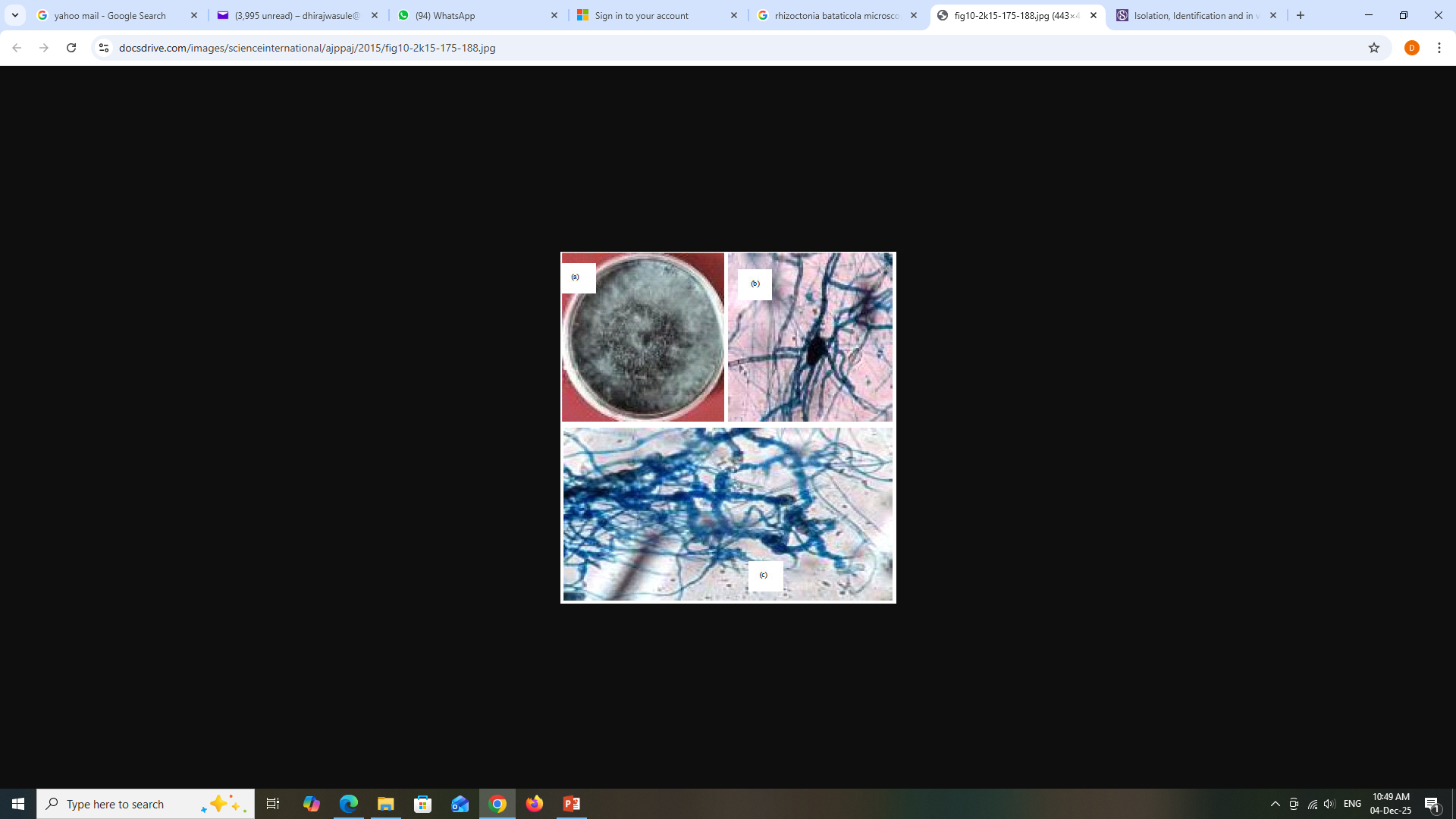


c


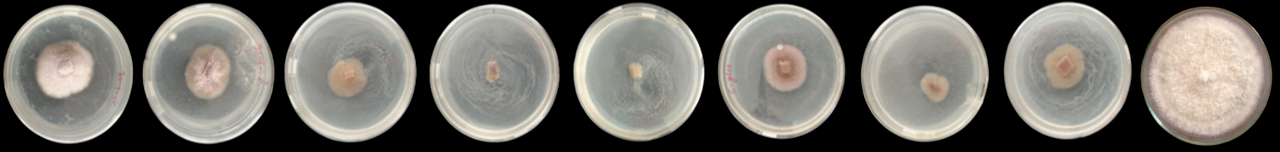


100 µg mL⁻¹

300 µg mL⁻¹

200 µg mL⁻¹

400 µg mL⁻¹

500 µg mL⁻¹

Chitosan

Bavistin

CuSO_4_.5H_2_O

Control

***Colletotrichum gloeosporioides***


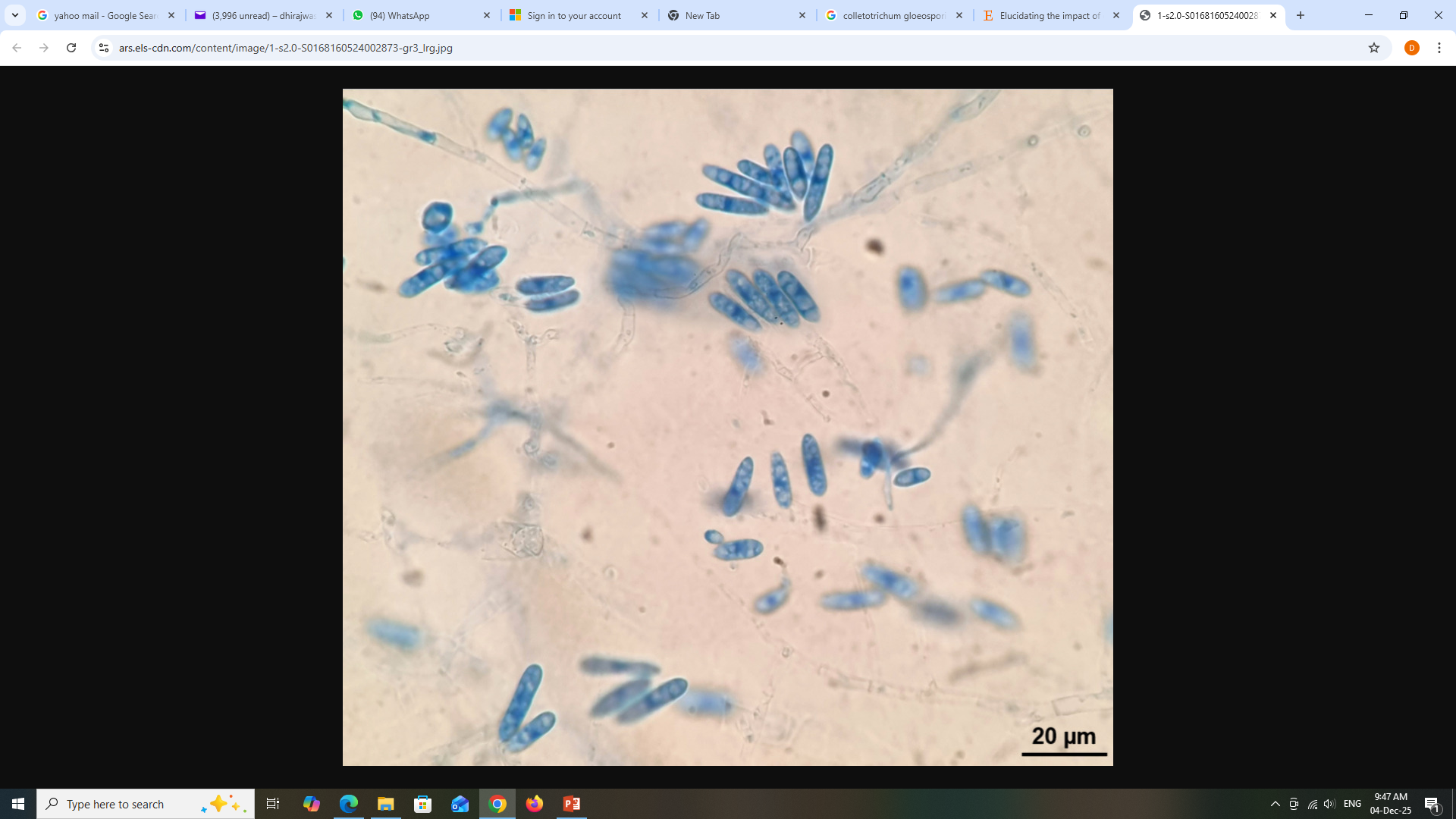


d


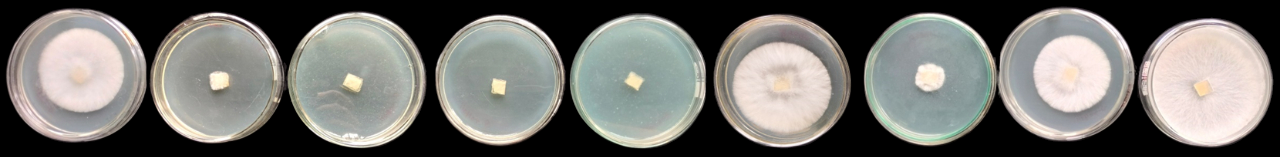


100 µg mL⁻¹

300 µg mL⁻¹

200 µg mL⁻¹

400 µg mL⁻¹

500 µg mL⁻¹

Chitosan

Bavistin

CuSO_4_.5H_2_O

Control

***Sclerotium rolfsii***

e


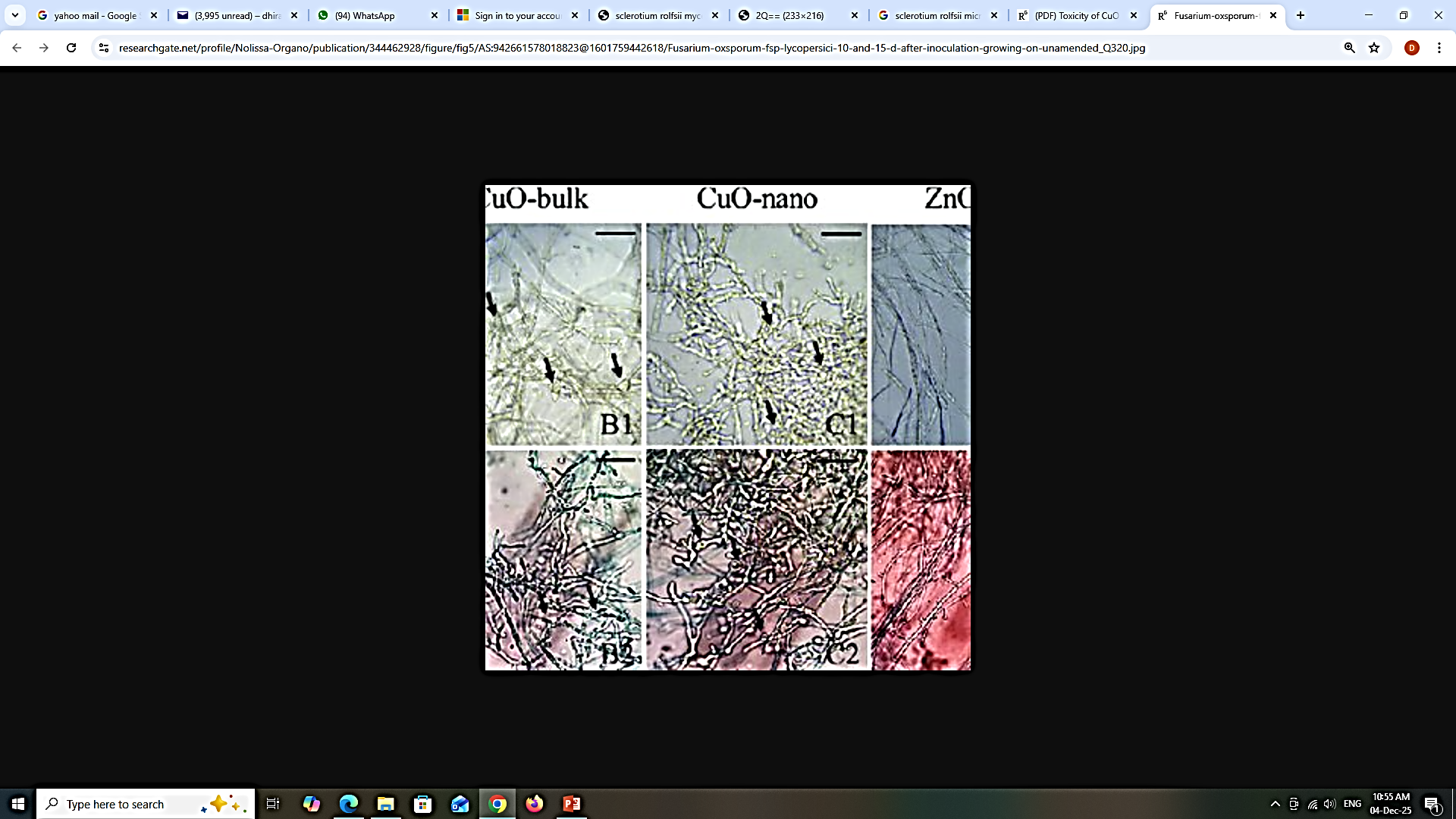


**Fig S1** Inhibitory effects of chitosan-copper nanoparticles (Cht-Cu NPs) on Fungal Pathogens a) *Colletotrichum ciceri* b) *Fusarium ciceri* c) *Rhizoctonia bataticola* d) *Colletotrichum gloeosporioides* e) *Sclerotium rolfsii*


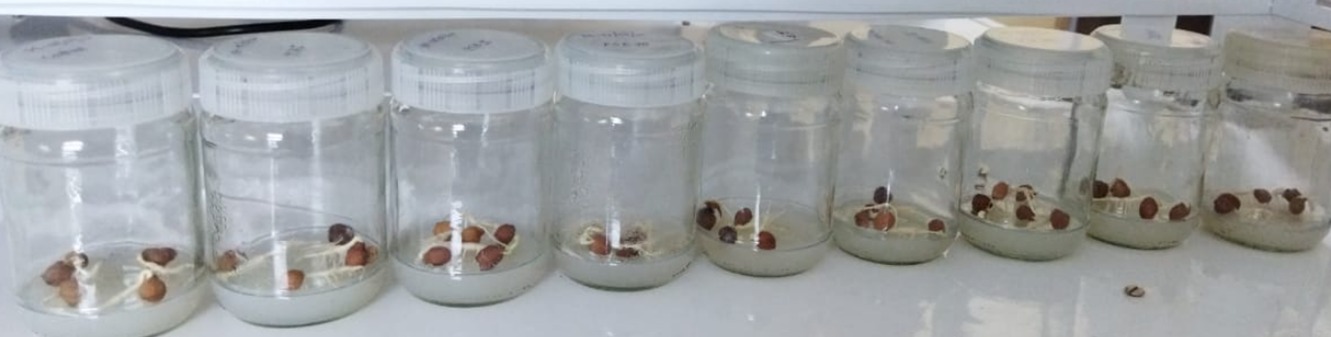


**Fig. S2.** *In vitro* seed germination assay for phytotoxicity evaluation. Surface-sterilized chickpea seeds cultured on half-strength Murashige and Skoog (½ MS) medium supplemented with CHT–Cu nanoparticles at different concentrations, showing normal germination without visible phytotoxic effects.


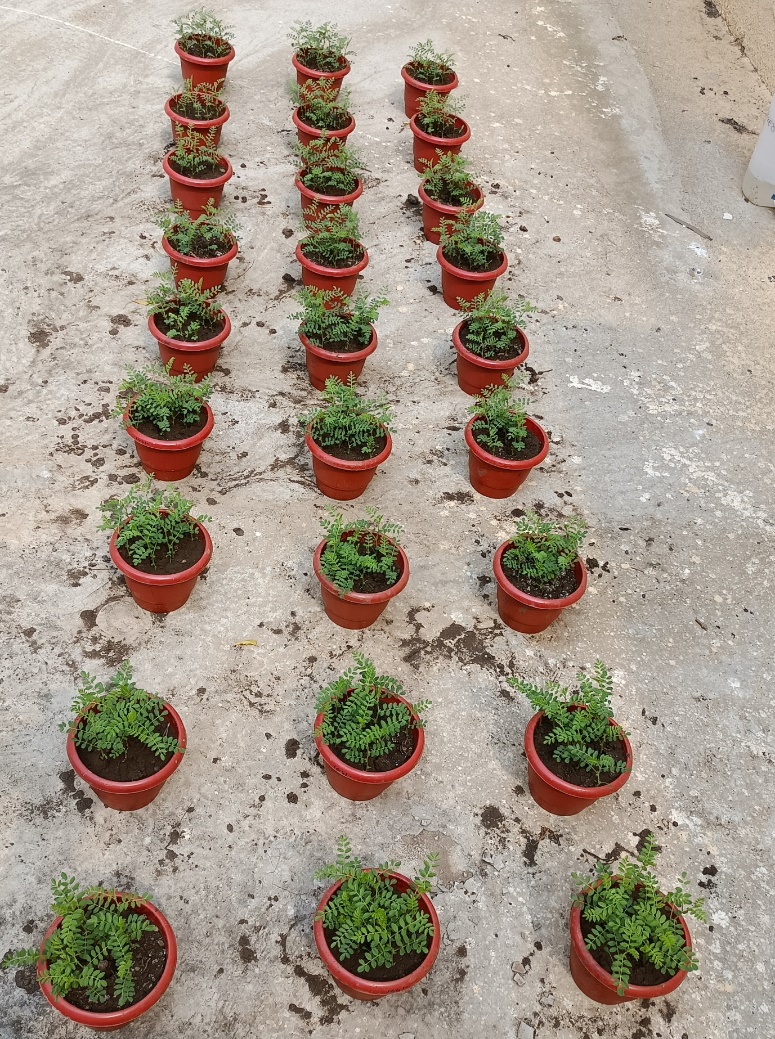


**Fig S3.** Pot experiment of transferred seedlings for phytotoxicity assessment of CHT–Cu nanoparticles in chickpea.

Chickpea seedlings 20 DAT in pots showing normal germination and uniform early seedling growth under greenhouse conditions.

**Table S1.** *In-vitro* cumulative release profile of copper from chitosan–copper nanoparticles (CHT–Cu NPs) showing time-dependent Cu release over 48–192 h, indicating sustained availability of Cu from the nanocomposite system.

| **Sr. No.** | **Time (h)** | **% Cu release (%)** |
| --- | --- | --- |
| 1 | 48 | 36.39 |
| 2 | 96 | 65.8 |
| 3 | 144 | 68.19 |
| 4 | 192 | 69.58 |

**Note**: Absorbance values are mean of three replications

## **Table S2.** Angular-transformed values (degrees) for *In vitro* antifungal activity of chitosan–copper nanoparticles (CHT–Cu NPs) against major chickpea and citrus pathogens at seven days after inoculation.

| **Treatments** | ***Colletotrichum ciceri*** | ***Fusarium ciceri*** | ***Rhizoctonia bataticola*** | ***Sclerotium rolfsii*** | ***Colletotrichum gloeosporioides*** |
| --- | --- | --- | --- | --- | --- |
| CHT Cu NPs @ 100 µg mL⁻¹ | 47.96 | 90.00 | 45.00 | 72.65 | 41.25 |
| CHT Cu NPs @ 200 µg mL⁻¹ | 67.79 | 90.00 | 57.49 | 90.00 | 46.50 |
| CHT Cu NPs @ 300 µg mL⁻¹ | 90.00 | 90.00 | 90.00 | 90.00 | 57.75 |
| CHT Cu NPs @ 400 µg mL⁻¹ | 90.00 | 90.00 | 90.00 | 90.00 | 90.00 |
| CHT Cu NPs @ 500 µg mL⁻¹ | 90.00 | 90.00 | 90.00 | 90.00 | 90.00 |
| Chitosan @ 4000 µg mL⁻¹ | 30.73 | 32.04 | 30.73 | 19.47 | 28.09 |
| Bavistin @ 500 µg mL⁻¹ | 43.07 | 45.00 | 38.36 | 42.34 | 38.08 |
| CuSO_4_.5H_2_O @ 280 µg mL⁻¹ | 36.94 | 38.15 | 36.94 | 19.47 | 34.55 |
| Control | – | – | – | – | – |

**Table S3. Broth-based determination of MIC of chitosan–metal nanocomposite against *Colletotrichum ciceri* at seven days after inoculation**

| **Treatment Concentration (µg mL⁻¹)** | **Fungal growth (+/−)** | **OD₆₀₀ / Visual turbidity** | **% Growth inhibition**  ***Colletotrichum ciceri*** | **MIC endpoint** |
| --- | --- | --- | --- | --- |
| CHT Cu NPs @ 100 µg mL⁻¹ | + | Moderate | 50 | - |
| CHT Cu NPs @ 200 µg mL⁻¹ | + | Low | 80 | - |
| CHT Cu NPs @ 300 µg mL⁻¹ | - | No visible growth | 100 | MIC |
| CHT Cu NPs @ 400 µg mL⁻¹ | - | No visible growth | 100 | - |
| CHT Cu NPs @ 500 µg mL⁻¹ | - | No visible growth | 100 | - |
| Chitosan @ 4000 µg mL⁻¹ | + | Moderate | 25 | - |
| Bavistin @ 500 µg mL⁻¹ | + | Moderate | 40 | - |
| CuSO_4_.5H_2_O @ 280 µg mL⁻¹ | + | Moderate | 30 | - |
| Control | + | High | 0 | - |

**Table S4. MFC determination (Subculturing from MIC assay) of chitosan–metal nanocomposite against *Colletotrichum ciceri* at seven days after inoculation on PDA**

| **Treatment Concentration (µg mL⁻¹)** | **Growth on PDA after subculture** | **Colony formation** | **MFC endpoint** |
| --- | --- | --- | --- |
| CHT Cu NPs @ 100 µg mL⁻¹ | Yes | Moderate | - |
| CHT Cu NPs @ 200 µg mL⁻¹ | ± | Few colonies | - |
| CHT Cu NPs @ 300 µg mL⁻¹ | No | Nil | MFC |
| CHT Cu NPs @ 400 µg mL⁻¹ | No | Nil | - |
| CHT Cu NPs @ 500 µg mL⁻¹ | No | Nil | - |
| Chitosan @ 4000 µg mL⁻¹ | Yes | Moderate | - |
| Bavistin @ 500 µg mL⁻¹ | Yes | Moderate | - |
| CuSO_4_.5H_2_O @ 280 µg mL⁻¹ | Yes | Moderate | - |
| Control | Yes | Profuse | - |

**Note:** All values are Mean ± SE

**Table S5. Broth-based determination of MIC of chitosan–metal nanocomposite against *Fusarium ciceri* at seven days after inoculation**

| **Treatment Concentration (µg mL⁻¹)** | **Fungal growth (+/−)** | **OD₆₀₀ / Visual turbidity** | **% Growth inhibition**  ***Fusarium ciceri*** | **MIC endpoint** |
| --- | --- | --- | --- | --- |
| CHT Cu NPs @ 100 µg mL⁻¹ | - | No visible growth | 100 | MIC |
| CHT Cu NPs @ 200 µg mL⁻¹ | - | No visible growth | 100 | - |
| CHT Cu NPs @ 300 µg mL⁻¹ | - | No visible growth | 100 | - |
| CHT Cu NPs @ 400 µg mL⁻¹ | - | No visible growth | 100 | - |
| CHT Cu NPs @ 500 µg mL⁻¹ | - | No visible growth | 100 | - |
| Chitosan @ 4000 µg mL⁻¹ | + | Moderate | 25 | - |
| Bavistin @ 500 µg mL⁻¹ | + | Moderate | 45 | - |
| CuSO_4_.5H_2_O @ 280 µg mL⁻¹ | + | Moderate | 33 | - |
| Control | + | High | 0 | - |

**Table S6. MFC determination (Subculturing from MIC assay) of chitosan–metal nanocomposite against *Fusarium ciceri* at seven days after inoculation on PDA**

| **Treatment Concentration (µg mL⁻¹)** | **Growth on PDA after subculture** | **Colony formation** | **MFC endpoint** |
| --- | --- | --- | --- |
| CHT Cu NPs @ 100 µg mL⁻¹ | No | Nil | MFC |
| CHT Cu NPs @ 200 µg mL⁻¹ | No | Nil | - |
| CHT Cu NPs @ 300 µg mL⁻¹ | No | Nil | - |
| CHT Cu NPs @ 400 µg mL⁻¹ | No | Nil | - |
| CHT Cu NPs @ 500 µg mL⁻¹ | No | Nil | - |
| Chitosan @ 4000 µg mL⁻¹ | Yes | Moderate | - |
| Bavistin @ 500 µg mL⁻¹ | Yes | Moderate | - |
| CuSO_4_.5H_2_O @ 280 µg mL⁻¹ | Yes | Moderate | - |
| Control | Yes | Profuse | - |

**Note:** All values are Mean ± SE

**Table S7. Broth-based determination of MIC of chitosan–metal nanocomposite against *Rhizoctonia bataticola* at seven days after inoculation**

| **Treatment Concentration (µg mL⁻¹)** | **Fungal growth (+/−)** | **OD₆₀₀ / Visual turbidity** | **% Growth inhibition**  ***Rhizoctonia bataticola*** | **MIC endpoint** |
| --- | --- | --- | --- | --- |
| CHT Cu NPs @ 100 µg mL⁻¹ | + | Moderate | 45 | - |
| CHT Cu NPs @ 200 µg mL⁻¹ | + | Moderate | 65 | - |
| CHT Cu NPs @ 300 µg mL⁻¹ | - | No visible growth | 100 | MIC |
| CHT Cu NPs @ 400 µg mL⁻¹ | - | No visible growth | 100 | - |
| CHT Cu NPs @ 500 µg mL⁻¹ | - | No visible growth | 100 | - |
| Chitosan @ 4000 µg mL⁻¹ | + | Moderate | 20 | - |
| Bavistin @ 500 µg mL⁻¹ | + | Moderate | 32 | - |
| CuSO_4_.5H_2_O @ 280 µg mL⁻¹ | + | Moderate | 30 | - |
| Control | + | High | 0 | - |

**Table S8. MFC determination (Subculturing from MIC assay) of chitosan–metal nanocomposite against *Rhizoctonia bataticola* at seven days after inoculation on PDA**

| **Treatment Concentration (µg mL⁻¹)** | **Growth on PDA after subculture** | **Colony formation** | **MFC endpoint** |
| --- | --- | --- | --- |
| CHT Cu NPs @ 100 µg mL⁻¹ | Yes | Moderate | - |
| CHT Cu NPs @ 200 µg mL⁻¹ | ± | Few colonies | - |
| CHT Cu NPs @ 300 µg mL⁻¹ | No | Nil | MFC |
| CHT Cu NPs @ 400 µg mL⁻¹ | No | Nil | - |
| CHT Cu NPs @ 500 µg mL⁻¹ | No | Nil | - |
| Chitosan @ 4000 µg mL⁻¹ | Yes | Moderate | - |
| Bavistin @ 500 µg mL⁻¹ | Yes | Moderate | - |
| CuSO_4_.5H_2_O @ 280 µg mL⁻¹ | Yes | Moderate | - |
| Control | Yes | Profuse | - |

**Note:** All values are Mean ± SE

**Table S9. Broth-based determination of MIC of chitosan–metal nanocomposite against *Colletotrichum gloeosporioides* at seven days after inoculation**

| **Treatment Concentration (µg mL⁻¹)** | **Fungal growth (+/−)** | **OD₆₀₀ / Visual turbidity** | **% Growth inhibition**  ***Colletotrichum gloeosporioides*** | **MIC endpoint** |
| --- | --- | --- | --- | --- |
| CHT Cu NPs @ 100 µg mL⁻¹ | + | Moderate | 35 | - |
| CHT Cu NPs @ 200 µg mL⁻¹ | + | Moderate | 46 | - |
| CHT Cu NPs @ 300 µg mL⁻¹ | + | Low | 65 | - |
| CHT Cu NPs @ 400 µg mL⁻¹ | - | No visible growth | 100 | MIC |
| CHT Cu NPs @ 500 µg mL⁻¹ | - | No visible growth | 100 | - |
| Chitosan @ 4000 µg mL⁻¹ | + | Moderate | 18 | - |
| Bavistin @ 500 µg mL⁻¹ | + | Moderate | 30 | - |
| CuSO_4_.5H_2_O @ 280 µg mL⁻¹ | + | Moderate | 25 | - |
| Control | + | High | 0 | - |

**Table S10. MFC determination (Subculturing from MIC assay) of chitosan–metal nanocomposite against *Colletotrichum gloeosporioides* at seven days after inoculation on PDA**

| **Treatment Concentration (µg mL⁻¹)** | **Growth on PDA after subculture** | **Colony formation** | **MFC endpoint** |
| --- | --- | --- | --- |
| CHT Cu NPs @ 100 µg mL⁻¹ | Yes | Moderate | - |
| CHT Cu NPs @ 200 µg mL⁻¹ | ± | Few colonies | - |
| CHT Cu NPs @ 300 µg mL⁻¹ | ± | Few colonies | - |
| CHT Cu NPs @ 400 µg mL⁻¹ | No | Nil | MFC |
| CHT Cu NPs @ 500 µg mL⁻¹ | No | Nil | - |
| Chitosan @ 4000 µg mL⁻¹ | Yes | Moderate | - |
| Bavistin @ 500 µg mL⁻¹ | Yes | Moderate | - |
| CuSO_4_.5H_2_O @ 280 µg mL⁻¹ | Yes | Moderate | - |
| Control | Yes | Profuse | - |

**Note:** All values are Mean ± SE

**Table S11. Broth-based determination of MIC of chitosan–metal nanocomposite against *Sclerotium rolfsii* at seven days after inoculation**

| **Treatment Concentration (µg mL⁻¹)** | **Fungal growth (+/−)** | **OD₆₀₀ / Visual turbidity** | **% Growth inhibition**  ***Sclerotium rolfsii*** | **MIC endpoint** |
| --- | --- | --- | --- | --- |
| CHT Cu NPs @ 100 µg mL⁻¹ | + | Low | 85 | - |
| CHT Cu NPs @ 200 µg mL⁻¹ | - | No visible growth | 100 | MIC |
| CHT Cu NPs @ 300 µg mL⁻¹ | - | No visible growth | 100 | - |
| CHT Cu NPs @ 400 µg mL⁻¹ | - | No visible growth | 100 | - |
| CHT Cu NPs @ 500 µg mL⁻¹ | - | No visible growth | 100 | - |
| Chitosan @ 4000 µg mL⁻¹ | + | Moderate | 7 | - |
| Bavistin @ 500 µg mL⁻¹ | + | Moderate | 40 | - |
| CuSO_4_.5H_2_O @ 280 µg mL⁻¹ | + | Moderate | 7 | - |
| Control | + | High | 0 | - |

**Table S12. MFC determination (Subculturing from MIC assay) of chitosan–metal nanocomposite against *Sclerotium rolfsii* at seven days after inoculation on PDA**

| **Treatment Concentration (µg mL⁻¹)** | **Growth on PDA after subculture** | **Colony formation** | **MFC endpoint** |
| --- | --- | --- | --- |
| CHT Cu NPs @ 100 µg mL⁻¹ | ± | Few colonies | - |
| CHT Cu NPs @ 200 µg mL⁻¹ | No | Nil | MFC |
| CHT Cu NPs @ 300 µg mL⁻¹ | No | Nil | - |
| CHT Cu NPs @ 400 µg mL⁻¹ | No | Nil | - |
| CHT Cu NPs @ 500 µg mL⁻¹ | No | Nil | - |
| Chitosan @ 4000 µg mL⁻¹ | Yes | Moderate | - |
| Bavistin @ 500 µg mL⁻¹ | Yes | Moderate | - |
| CuSO_4_.5H_2_O @ 280 µg mL⁻¹ | Yes | Moderate | - |
| Control | Yes | Profuse | - |

**Note:** All values are Mean ± SE

**Table S13. Phytotoxic impact of chitosan–metal nanocomposite chickpea grown under *in-vitro* conditions.**

| **Treatment Concentration (µg mL⁻¹)** | **Germination percentage** | **Seedling vigor index** |
| --- | --- | --- |
| CHT Cu NPs @ 100 | 93.3 | 740.67 |
| CHT Cu NPs @ 200 | 93.3 | 744.00 |
| CHT Cu NPs @ 300 | 93.3 | 746.00 |
| CHT Cu NPs @ 400 | 100.0 | 796.67 |
| CHT Cu NPs @ 500 | 93.3 | 740.67 |
| CHT Cu NPs @ 600 | 93.3 | 744.00 |
| CHT Cu NPs @ 700 | 93.3 | 746.00 |
| CHT Cu NPs @ 800 | 86.7 | 690.00 |
| Control | 93.3 | 744.00 |
